# Supplementary material for: No impact of gestational diabetes mellitus on pregnancy complications in women with PCOS, regardless of GDM criteria used
Source: PLoS One. 2021 Jul 23;16(7):e0254895. doi: 10.1371/journal.pone.0254895 (PMC8301673; doi:10.1371/journal.pone.0254895)
Supplement: S1 Table — (DOCX) [file pone.0254895.s001.docx]

**Supporting information**

**S1 Table.** **GDM in accordance with the Norwegian 2017-criteria in 685 women with PCOS.**

|  |  | **Non-GDM** | **GDM** | **p-value** |
| --- | --- | --- | --- | --- |
|  |  | 500 (73 %) | 186 (27 %) |  |
| **Randomization** | **Metformin, N (%)** | 241 (48) | 85 (46) | 0.454 |
|  |  |  |  |  |
| **Baseline data** | **Age, years** | 29.4 ± 4.2 | 30.6 ± 4.6 | **0.002** |
|  | **BMI, kg/m2** | 27.6 ± 5.7 | 30.8 ± 7.2 | **<0.001** |
|  | **Weight, kg** | 77.8 ± 16.7 | 85.4 ± 20.9 | **<0.001** |
|  | **Nulliparous** | 291 (58) | 105 (56) | 0.53 |
|  | **Comorbidity** | 227 (45) | 101 (54) | 0.038 |
|  | **Smoking** | 24 (4.8) | 12 (6.5) | 0.40 |
|  |  |  |  |  |
| **Maternal outcome** | **Hypertension, debut in pregnancy** | 24 (4.8) | 9 (4.8) | 0.23 |
|  | **Preeclampsia** | 31 (6.2) | 12 (6.5) | 0.90 |
|  | **Weight gain, kg *** | 10.8 ± 4.8 | 8.6 ± 5.6 | **<0.001** |
|  |  |  |  |  |
| **Neonatal outcome** | **Birth weight, g** | 3532 ± 613 | 3540 ± 586 | 0.88 |
|  | **Birth weight, z-score** | -0.05 ± 1.01 | -0.01 ± 1.08 | 0.64 |
|  | **Gest. age, days** | 277 ± 20 | 275 ± 19 | 0.39 |
|  | **SGA/LGA** | 56 (11)/39 (8) | 18 (10)/22 (12) | 0.24 |

Clinical characteristics and pregnancy outcome for women with and without gestational diabetes (GDM), classified after the Norwegian 2017 criteria. Values given as mean ± SD or N (%) as appropriate. * N= 626. HT hypertension, PE preeclampsia, SGA small for gestational age, LGA large for gestational age.

Values given as mean ± SD or N (%) as appropriate. SGA small for gestational age, LGA large for gestational age. *One patient receiving metformin was treated with insulin due to GDM diagnosed using the new WHO 2013 criteria, but she did not have GDM using the Norwegian 2017 criteria.
