# Supplementary material for: No impact of gestational diabetes mellitus on pregnancy complications in women with PCOS, regardless of GDM criteria used
Source: PLoS One. 2021 Jul 23;16(7):e0254895. doi: 10.1371/journal.pone.0254895 (PMC8301673; doi:10.1371/journal.pone.0254895)
Supplement: S2 Table — (DOCX) [file pone.0254895.s002.docx]

**Supporting information**

**S2 Table.** **Pregnancy complications in patients treated with metformin and placebo, women with and without GDM (according to Norwegian 2017 criteria).**

|  | **GDM** | | | **Non-GDM** | | |
| --- | --- | --- | --- | --- | --- | --- |
|  | **Metformin** | **Placebo** | **p-value** | **Metformin** | **Placebo** | **p-value** |
| **N** | 85 | 101 |  | 242 | 258 |  |
| **Hypertension** | 6 (7) | 3 (3) | 0.15 | 11 (5) | 13 (5) | 0.71 |
| **Preeclampsia** | 6 (7) | 6 (6) | 0.76 | 14 (6) | 17 (7) | 0.71 |
| **Late miscarriage/ preterm birth** | 1 (1) | 10 (10) | 0.012 | 11 (5) | 26 (10) | 0.018 |
| **SGA/LGA** | 4 (5)/11 (13) | 14 (14)/11 (11) | 0.10 | 30 (13)/20 (8) | 26 (10)/19 (7) | 0.66 |
| **Birth weight** | 3639 ± 501 | 3442 ± 685 | 0.028 | 3543 ± 521 | 3538 ± 644 | 0.92 |
| **Birth weight, z-score** | 0.10 ± 1.09 | -0.11 ± 1.07 | 0.19 | -0.12 ± 1.05 | 0.02 ± 0.97 | 0.14 |
| **Gest. age, days** | 278 ± 10 | 273 ± 24 | 0.050 | 278 ± 16 | 276 ± 23 | 0.31 |
| **Matern. weight gain, kg** | 7.6 ± 5.3 | 9.4 ± 5.7 | 0.037 | 9.5 ± 6.0 | 12.2 ± 8.0 | **<0.001** |
| **Insulin treatment** | 7 (8) | 13 (13) | 0.31 | 1 (0.04)* | 0 | 0.31 |

Values given as mean ± SD or N (%) as appropriate. SGA small for gestational age, LGA large for gestational age. *One patient receiving metformin was treated with insulin due to GDM diagnosed using the new WHO 2013 criteria, but she did not have GDM using the Norwegian 2017 criteria.
