# Supplementary material for: No impact of gestational diabetes mellitus on pregnancy complications in women with PCOS, regardless of GDM criteria used
Source: PLoS One. 2021 Jul 23;16(7):e0254895. doi: 10.1371/journal.pone.0254895 (PMC8301673; doi:10.1371/journal.pone.0254895)
Supplement: S3 Table — a. GDM diagnosed any time in pregnancy. b. GDM diagnosed in early pregnancy (at inclusion). (DOCX) [file pone.0254895.s003.docx]

**Supporting information**

**S3 Table.** **Comparison of the prevalence between the different GMD classifications.**

1. GDM diagnosed any time in pregnancy

|  |  | **GDM according to WHO 2013** | |  |
| --- | --- | --- | --- | --- |
|  |  | **No** | **Yes** | **Total** |
| **GDM according to WHO 1999** | **No** | 371 | 135 | 506 (74 %) |
|  | **Yes** | 42 | 138 | 180 (26 %) |
|  | **Total** | 413 (60 %) | 273 (40 %) | 686 |

Values given as N, except for total N (%). McNemar’s test p<0.001

1. GDM diagnosed in early pregnancy (at inclusion)

|  |  | **GDM according to WHO 2013** | |  |
| --- | --- | --- | --- | --- |
|  |  | **No** | **Yes** | **Total** |
| **GDM according to WHO 1999** | **No** | 590 | 105 | 695 (90 %) |
|  | **Yes** | 23 | 54 | 77 (10 %) |
|  | **Total** | 613 (80 %) | 159 (20 %) | 772 |

Values given as N, except for total N (%). McNemar’s test p<0.001
