# Supplementary material for: No impact of gestational diabetes mellitus on pregnancy complications in women with PCOS, regardless of GDM criteria used
Source: PLoS One. 2021 Jul 23;16(7):e0254895. doi: 10.1371/journal.pone.0254895 (PMC8301673; doi:10.1371/journal.pone.0254895)
Supplement: Attachment — Submitted filename: Response to reviewers.PlosOne_2_mars2021.docx [file pone.0254895.s004.docx]

Dear Editor,

Thank you for considering our manuscript “The impact of gestational diabetes mellitus on pregnancy complications in women with PCOS” for publication in PLOS ONE.

We thank the Editor and the Reviewers for their time and effort spent on reviewing the manuscript. Below are the responses to the comments by each of the reviewers.

**Reviewer #1:** This is an interesting paper, quite relevant to what are PLOS One's publication criteria. It is becoming increasingly recognized that in GDM, maternal weight gain during the pregnancy is a major determinant of outcome, and may be more than important than hyperglycaemia.
The authors quite rightly make the point that no weight data was collected during the HAPO study, and is perfectly possible that the increased glucose levels associated with adverse outcomes may have been a very good correlate of increased body mass.
The authors have clearly demonstrated that in an (largely) obese and almost always insulin resistant group of pregnant women, given the label of PCOS, whatever level of new onset hyperglycaemia in pregnancy is found, then the outcome is no different. It is perhaps not surprising therefore then in those who were randomized to metformin there was little if any significant difference.
The major weakness of the study, acknowledged by the authors, is no control group of course.
Nonetheless I feel strongly that these findings add more to our understanding of the importance of non-glycaemic factors in GDM.

**Answer:** Thank you for thorough reading of our manuscript and your positive review.

We appreciate your comment on the importance of gaining more knowledge on the factors not related to hyperglycemia in women with GDM, which we now believe to be more important than previously regarded.

**Reviewer #2:** Here is a list of specific comments. Note: line and page numbering in reviews and comments is based on ruler applied in Editorial Manager-generated PDF.

1. Page 7, lines 133–135: Per lines 106–107, data from the OGTT test at baseline were also available. I suggest including it in the Baseline Data section.

**Answer:** Thank you for your suggestion. We agree and have included the information in the Baseline Data section.

2. Page 8, lines 149–153: I did not understand what the z-scores represent (i.e., z-scores of what). I assumed it was birth weight. Did you mean to state the following? ‘For all live births after gestational week 24, a gestational age- and gender-adjusted birth weight was calculated based on Niklasson’s standard values from a large Swedish population. The z-score transformed birth weight express the deviation between observed birth weight and the Swedish population mean birth weight, adjusted for sex and gestational age at birth.’
**Answer:** Thank you for pointing out some unclarity in our text. We have now adjusted the text as suggested.

3. Page 9, lines 160–162: In multivariable regressions, I suggest elaborating how determinants were selected into the regressions.
**Answer:** Thank you for your correct notification. This is now included in the Statistics section.

4. Page 10, line 183: Between the first and second paragraphs in the GDM and Baseline Characteristics section, it might be interesting to see a comparison between the WHO 1999 criteria and the WHO 2013 criteria using a 2x2 table with McNemar’s test statistics. Please feel free to add the Norwegian 2017 criteria to the mix but it certainly increases the number of comparisons.
**Answer:** We agree and have added this 2x2 table in the Supplemental file (Suppl. Table 3).

5. Page 10, lines 195–198: I suggest specifying which correlation statistic was used (i.e., what R represented) in the Statistical Analyses section.
**Answer:** Thank you for the notification of omitted important information. This is now included in the Statistics section

6. Page 11, line 221: In the logistic regressions in Table 2b, what was the rationale for not including GDM? Up to this point, all comparisons were centered around GDM. It would be interested to see how GDM associated with late miscarriage.
**Answer:** For this regression analysis, we wanted to include the glucose data (fasting glucose and 2h-glucose) instead of the dichotomized parameter GDM. This was possible since the glucose data were collected at one time point in pregnancy. We found it more interesting to analyze if fasting glucose was more important than 2h-glucose, or vice versa, than using the “double” dichotomized parameter GDM. Using continuous data (fasting and 2 h glucose) instead of dichotomized data (GDM) in general also increase the statistical power and possibility to identify possible biologic associations. For the regression analysis of birth weight, however, we had glucose data from different time points in the pregnancy, and this was not possible. For this analysis, we had to use the dichotomized parameter GDM sometime in the pregnancy and perform two analyses, one for each GDM criterion (WHO 1999 and WHO 2013).

7. Page 13, line 257: The analyses in the Metformin Treatment in Women with and without GDM sections may be biased. The analyses were separated by GDM, a post Metformin variable. Analyses stratified by a post-baseline variable would require a more careful analytic approach or require a cautious note regarding the potential bias the analyses may infer.
**Answer**: For the analyses of parameters registered late on pregnancy or at birth like preeclampsia and, we agree with the reviewer. There is a potential bias when looking at the GDM and non-GMD group separately, where we cannot be sure if the treatment with Metformin during second and third trimester influence the later development of GDM. However, we did not find any effect of Metformin regarding the glucose levels or the prevalence of GDM. There is still a theoretically risk for Metformin influencing which women who develop GDM, however, we hold this as less possible. In this regard, it is interesting that the observed effects of metformin are similar for both groups (GDM and non-GMD). However, for birth weight, this was accounted for in the regression analyses where both Metformin and GDM status were included (please see page 12).

In addition, regarding the risk for late miscarriage and preterm birth combined, the risk for this potential bias is even less likely, since most women with GDM in this group were diagnosed at inclusion. Of the 6 women diagnosed with GDM later in pregnancy after any criteria, three had received Metformin and three placebo treatment. We have not included these details in the paper, but we can do so if indicted by Editor.

8. Table 2b:
(8a) For both univariable and multivariable logistic regressions, I suggest reporting odds ratios and their 95% confidence intervals (instead of B). Also, I suggest replacing “multivariate” with ‘multivariable’.
**Answer:** Thank you for the correct notification on parameters reported. The Table is now updated.

(8b) Lines 569–570: For continuous variables, t-test was not appropriate in this case. I suggest reporting p-values using univariable logistic regressions. Note that the univariable logistic regressions can be used for dichotomous variables as well where p-values should be the same as the p-values from chi-square tests.
**Answer:** Thank you for the correct notification on parameters reported. We have now reported the p-values using univariable logistic regression, in addition to reporting odds ratio and confidence interval (except for one parameter where confidence interval did not give any sense – please see revised Table legend).

9. Table 3:
(9a) Please clarify what R represented.
**Answer:** Thank you for the correct notification on parameters reported. R represented the Pearson correlation coefficient but is now replaced with beta and confidence interval from univariable linear regression.

(9b) Instead of beta/t, I suggest reporting regression coefficients (betas) and their 95% confidence intervals.

**Answer:** Thank you for the correct notification on parameters reported. The Table is now updated.

**Reviewer #3:** I have reviewed this submission by Fougner et al. The study included three cohorts of PCOS women randomized in three different trials in three different eras to receive metformin versus placebo. The study is comparing the pregnancy outcomes between women with and without GDM. The study has significant issues that question it's validity.
Major points
1. The study title indicates that the focus of the paper is on pregnancy outcomes. However, the manuscript includes lengthy details describing the difference in women's prevalence and characteristics with GDM using three different criteria. In my view, this could be the main focus of the manuscript. The title should reflect this part of the study.

**Answer:** We hold the view that the title should reflect our hypothesis (the reason for performing the study) and/or the most important observation. Our main focus of this study was to evaluate the impact of GDM on pregnancy complications. However, to do this in detail, we had to classify the patients according to the criteria for GDM which has recently been changed. Our opinion is that an analysis with only old and now outdated criteria would not be sufficient. With our almost complete glucose data we choose to evaluate this question using the newer GDM criteria now widely used, in addition to the older criteria used at the time of the studies. At the same time, we had the opportunity to compare both the old and the new GDM criteria to pregnancy outcome in this large, well defined patient cohort and also to evaluate the possible difference between the GDM criteria. We state that pregnancy outcome is the most important part of our publication, and that the title therefore is correct. We consider the prevalence of GDM and the clinical characteristics of women with GDM according to the different criteria more to be a necessary and interesting introduction to analysis of the main hypothesis in this work. We are not aware that any data similar to our data on the missing association between glucose levels and pregnancy outcomes has been presented before. That is the novelty of the present study and not the difference in GDM according to different criteria. However, we have adjusted the title, now indicating the use of different CGM criteria.

2. The authors did not outline what the three diagnostic criteria in the manuscript are. In particular – what is the Norwegian criteria?
**Answer:** Information on all GDM criteria used were, and still are, stated in the Methods section, under the subsection GDM Classification. No adjustments of the manuscript have been made at this point.

3. There was no standardized management protocol for women with GDM. Hence, it cannot be assumed that all women were treated to a unified target. The glycaemic management of women with GDM is quite critical to pregnancy outcomes.
**Answer:** All women were classified as having GDM and treated for GDM according to national standard and guidelines at the time. Thus, none were deprived of standard GDM treatment.

We agree that the management of GDM are important, but our data on missing association between glucose levels and pregnancy outcomes in a well-defined cohort of pregnant women with PCOS challenge the view that glycaemic management is most critical.

4. The authors used different criteria and applied them to the whole cohort and then classified them as GDM or no GDM based on three different criteria. However, this is a messy and untidy way to define GDM. It essentially means that some women classified with GDM using one classification were essentially classified as Normal Glucose Tolerant (NGT) during pregnancy and did not receive any treatment.
**Answer:** All women were classified as having GDM and treated for GDM according to national guidelines, thus, no women were deprived of standard GDM treatment. For almost all included women, the same GDM classification and clinical practice for management were used (WHO 1999 criteria). Only very few of the women in the last study (PregMet 2), that is only the last 10-15 included women in Iceland, were classified according to the new WHO 2013 criteria. Of them, only 3 women were diagnosed with GDM after new criteria, and only one patient received insulin treatment (from pregnancy week 36).

We hold that the fact that some women in these studies, according to criteria developed after their pregnancy, would have been classified as having GDM today, or vice versa, do not change the importance of these evaluations. Since almost all women in these studies were included prior to implementation of the newer GDM criteria, we also had the possibility to evaluate the group of women that did not get a GDM diagnose in pregnancy (according to the old GDM criteria) and therefore no treatment, but would have been diagnosed with GDM today, according to the new criteria. In this way, we argue that differences in gestational age and gestational weight gain is a result of the treatment and not the condition itself.

5. Glycaemic control is the most critical factor in the pregnancy outcomes of GDM. There was neither unified management protocol nor consistency in the women's classification during pregnancy- hence the pregnancy outcomes are not valid.
**Answer:** We agree that the common wisdom hold by most physicians is that glycemic control is the most critical factor influencing on outcomes in GDM. However, this is what our data and our paper challenge. Regarding diagnose and management of GDM, see answers above.

6. Hence, If the authors would like to proceed with this paper, I will advise them to drop the pregnancy outcomes and only report the prevalence of GDM using three different criteria. Alternatively, they can do a re-analysis using the actual classification of the women during pregnancy.
**Answer:** With all respect, we do not agree with this suggestion. We find it important to study the impact of glucose levels and the presence of gestational diabetes on pregnancy outcomes in a well-defined patient cohort. The fact that our data challenge the previous opinion that glucose levels are the most critical factor influencing outcome, makes it important to publish our data and make the findings available.

In fact, the analyses of GDM according to the WHO 1999 criteria is the analyses using the actual classification of the women used during pregnancy, as the Reviewer suggest, please see the answer to pt. 4 above. Together with other studies, our data might lead to an increased focus on the total risk profile of the pregnant women and not only on the glucose levels.

Minor points
1. Under the abstract, the authors wrote, "having GDM according to newer criteria correlated to increased maternal age and BMI (p<0.001), while GDM already in the first trimester associated with increased risk for late miscarriage (p<0.01)." What does this mean?
**Answer:** Having GDM anytime during pregnancy correlated to increased maternal age and BMI. Having GDM already at inclusion in the first trimester associated with risk of late miscarriage. For the women with late miscarriage, only glucose data from the inclusion in the late first trimester was available. We have rephrased the sentences and hope this is clearer now.

2. In the introduction, the authors wrote "IFD diabetes atlas" is IDF- diabetes atlas.
**Answer:** Thank you for pointing out our misspelling. This has been corrected.

3. They stated, "The HAPO study demonstrated a positive association between glucose levels and the proportion of neonates with a birth weight above 4000 g". This is not correct; the HAPO showed correlation with birth weight > 90th percentile.

**Answer:** The original publication from the HAPO study, ref. 15, found an association with birth weight above 4000 g, but a newer post hoc analysis of the HAPO material correctly found a correlation with birth weight >90^th^ percentile. This is already discussed in the Discussion section, and with ref. 31.

4. The HAPO trial reported on Gestational weight gain. The authors can refer to some of their publications.
**Answer:** We have both read and referred to several of the HAPO publications. However, we have not been able to find any publication referring data on maternal gestational weight gain. We will be happy to discuss and refer a publication from this large and important study reporting weight data if there are publications that we have not found. Please, see also the comment made by reviewer #1 on this topic.

5. The authors should report all the p-values and not only mention ns.

**Answer:** We agree and have added the exact p-value to the Table.
