# Supplementary material for: No impact of gestational diabetes mellitus on pregnancy complications in women with PCOS, regardless of GDM criteria used
Source: PLoS One. 2021 Jul 23;16(7):e0254895. doi: 10.1371/journal.pone.0254895 (PMC8301673; doi:10.1371/journal.pone.0254895)
Supplement: Attachment — Submitted filename: Response to reviewers.PlosOne_2_juni2021.docx [file pone.0254895.s005.docx]

Dear Editor,

Thank you for considering our manuscript “No impact of gestational diabetes mellitus on pregnancy complications in women with PCOS, regardless of GDM criteria used” for publication in PLOS ONE.

We thank the Editor and the Reviewers for their time and effort spent on reviewing the manuscript. Below are the responses to the comments by each of the reviewers.

**Reviewer #1:** Having reviewed the submission carefully, and the comments of reviewer 3 in particular, I do still feel that this study should be published. I think it would be a better paper if it was presented as a study of different classifications of GDM, but the authors are being perfectly reasonable in sticking to their original hypothesis. I think they have justified this decision, and as such satisfy, in their revisions, much of the original concerns. I do have one or two major issues though.
I apologise to the Editor and the authors. The HAPO study does present data on weight in pregnancy in GDM, but not weight gain. Hyperglycaemia and Adverse pregnancy outcome (HAPO) study: Association with maternal body mass index BJOG 2010 117(5): 577-84***.***  
This concludes 'higher maternal BMI, independently of maternal glycaemia is strongly associated with increased frequency of pregnancy complications'.
Secondly, The Hyperglycaemia and Adverse Pregnancy outcome Study. Association of GDM and obesity with pregnancy outcomes. Diabetes Care 2012 35(4): 780-786 which concludes 'both maternal GDM and obesity are independently associated with adverse pregnancy outcomes.
These references should be included.

***Answer:*** *We agree that data on maternal weight measured in third trimester have been published in later publications from the HAPO study. The study published in Diabetes Care 2012 was already included and discussed in the Discussion section (reference 31). We have now included also the HAPO publication in BJOG 2010 (new reference 32).*

I think is incorrect to state that metformin use should be suspended until placebo controlled RCTs are performed in GDM, this isn't going to happen.

***Answer:*** *We agree that this maybe is not going to happen, at least worldwide. However, our opinion is still that further studies, RCTs, on the efficacy of metformin is necessary before treating pregnant women with a medication passing the placenta and being present in fetal blood at the same level as in the mother. Several studies suggest that metformin-exposed children have higher BMI and more often overweight during childhood (Hanem JCEM 2018, Rowan BMJ Open Diab Res Care 2018). RCTs on the efficacy of metformin treatment in pregnant women are now only available in women with PCOS, women with hypertension, women with insulin resistance prior to pregnancy and obese women. In these studies, despite the high risk for GDM in these women, metformin treatment did not influence the glucose homeostasis nor reduced the incidence of GDM.*

Finally as a minor point there is a tangle in the use of English, line 164 it should say parameters that either were instead of either was and in line 166 it should say only one was rather than only one were.

***Answer:*** *We are sorry for the grammatical errors. Thank you for noticing, they are now corrected.*

**Reviewer #4:** The authors in this subanalysis of three RCTs concluded that, in pregnant women with PCOS, the GDM diagnosis did not increase the risk of late pregnancy complications. This data is surprising and it may be explained in only two ways: the risk of pregnancy complications in women with PCOS is per se so high that it is difficult to increase it and/or pregnant women with PCOS have different response to GDM in comparison with non-PCOS populations.

***Answer:*** *We agree that our data could be surprising, however, as discussed in the Introduction and the Discussion sections other studies have also suggested that underlying factors can be more important than the glucose level per se. We agree that the two mentioned explanations both are possible. We cannot know whether women with PCOS have a “different GDM” or a different response to GDM compared to women without PCOS, since all women in these studies have PCOS. But as PCOS is a common condition and the prevalence of GDM is markedly increased in PCOS, these women probably constitute a significant proportion of all women with GDM. This is discussed at the end of the Discussion section.*

The introduction section should be reduced in length of about 30%.

***Answer:*** *We feel that all parts of the introduction now is necessary to explain our rationale for this substudy. The length of the introduction has not been commented by the other three reviewers. However, we will try to shorten the section if the Editor find it best.*

The authors define the GDM “criteria” using only glucose values at OGTT irrespective from gestational age (when assays were taken). Please complete the methods section and discuss.

***Answer:*** *As described in the Methods section, an OGTT was performed after protocol at two or three different visits during the pregnancy. We classified GDM using the glucose levels at each time point. It is correct that we use the same glucose levels at the different study visits.*

Have you excluded patients with an abnormal OGTT at 12 weeks? Please clarify and discuss.
Can you give data on pregnancy complications on both populations (with GDM and pregestational diabetes)?

***Answer:*** *Patients with known pregestational diabetes and with fasting glucose ≥7 mmol/l at screening were not included in the randomized studies (exclusion criteria – please see the referred original publications). Women included in the RCTs that had an abnormal OGTT at the inclusion visit at appr. 12 weeks were not excluded in this substudy. They were registered as having early GDM. For data on pregnancy complications in the population with abnormal versus normal OGTT at 12 weeks, please see the sections “GDM at inclusion” and “Early versus late GDM”.*

Please clarify if all patients had a natural pregnancy or ART pregnancy (including medications/drugs).

***Answer:*** *Of the 722 women included in this substudy, 312 women (43 %) had a spontaneous pregnancy, while the remaining 410 women had received assistance. Detailed information on this is given in the original publication for each RCT.*

Please clarify where the patients were followed. One of the main concerns in terms of pregnancy complications in infertile e/o PCOS patients is the Hospital for obstetric management.

***Answer:*** *The women were followed with regular clinical and study visits at the Department of Gynaecology and Obstetrics of each study hospital. Detailed information on this is given in the original publication for each RCT.*

In my opinion, the analysis should be restricted to PCOS patients who did not use metformin (non-randomized to metformin) in order to avoid a potential confounder. Data on metformin should be analyzed as secondary aim in another different population and substudy.

***Answer:*** *Metformin could be a potential confounder. However, we did not find any difference between the women treated with metformin or not, except for the combined endpoint late miscarriage and preterm birth. Particularly, there were absolutely no difference in glucose levels or GDM incidence between the groups receiving metformin or placebo. This question is also taken into account with the analyses done separately for the two groups (metformin and placebo), see table 5, and also in the regression analyses where randomization was included as variable. Also, for the analyses of early GDM, the women had not yet started with study medication.*

The authors should clarify how the maternal weight gain was calculated and analyzed as risk factor. The increase in body weight throughout pregnancy is physiologic and different in obese and non-obese women (suggestions of the international guidelines).

***Answer:*** *As described in the Methods section, Pregnancy outcome, maternal weight gain was calculated as the difference between maternal weight at the visit in pregnancy week 36 and the maternal weight at the inclusion visit in week 12, given in kilogram. We did not relate the weight gain in kg to the IOM guideline according to prepregnant maternal BMI.*
